# Renal osteodystrophy: A historical review of its origins and conceptual evolution

**DOI:** 10.1016/j.bonr.2022.101641

**Published:** 2022-11-24

**Authors:** Garabed Eknoyan, Sharon M. Moe

**Affiliations:** a The Selzman Institute of Kidney Health, Section of Nephrology, Department of Medicine, Baylor College of Medicine, Houston, TX, USA; b Division of Nephrology and Hypertension, Indiana University School of Medicine, Indianapolis, IN, USA

**Keywords:** Renal osteodystrophy, Rickets, Osteoporosis, CKD-MBD, Bone

## Abstract

Long considered an inert supporting framework, bone studies went neglected until the 17th century when they began as descriptive microscopic studies of structure which over time progressed into that of chemistry and physiology. It was in the mid-19th century that studies evolved into an inquisitive discipline which matured into the experimental investigation of bone in health and disease in the 20th century, and ultimately that of molecular studies now deciphering the genetic language of bone biology. These fundamental studies were catalyzed by increasing clinical interest in bone disease. The first bone disease to be identified was rickets in 1645. Its subsequent connection to albuminuric patients reported in 1883 later became renal osteodystrophy in 1942, launching studies that elucidated the functions of vitamin D and parathyroid hormone and their role in the altered calcium and phosphate metabolism of the disease. Studies in osteoporosis and renal osteodystrophy have driven most recent progress benefitting from technological advances in imaging and the precision of evaluating bone turnover, mineralization, and volume. This review exposes the progress of bone biology from a passive support structure to a dynamically regulated organ with vital homeostatic functions whose understanding has undergone more revisions and paradigm shifts than that of any other organ.

## Highlights

• The study of bone in health and disease went neglected until the 17th century.
• Research began in bone structure evolving to chemistry and then physiology.
• Studies of rickets, renal osteodystrophy, and osteoporosis catalyzed discoveries.
• Technological advances were instrumental in its recent progress.
• Bone has evolved from a passive support structure to a vital endocrine organ.

## 1 Introduction

**Fig. 1:**
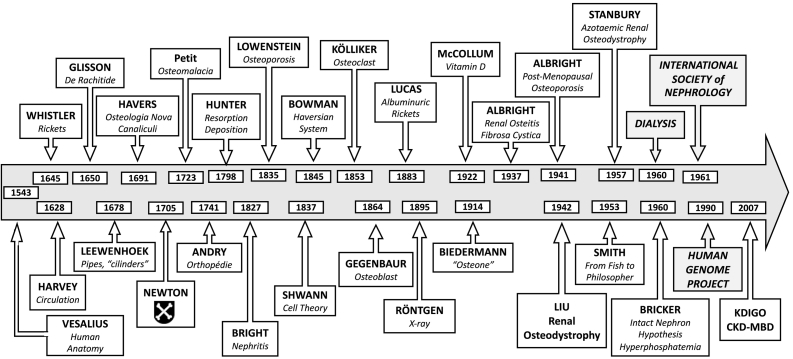
Timeline of the evolving understanding of the relation of bone (boxes above arrow) to that of kidney function (boxes below arrow). The name of principal contributors is shown in bold letters and their contribution in italics.

For most of its recorded history bone has gone as a cryptic organ of greater elegiac, artistic, ornamental, reliquary, weaponry, and poetic interest than of medical inquiry. Extant medical records indicate that whereas physicians of old were impressed by the hardness of bones, appreciated their structural importance in supporting the human body, and valued their role in providing protection to softer vital organs they did not consider bones deserving of study. These general notions prevailed well into the 16th century as recorded in 1543 by Andreas Vesalius (1514–1564) in his milestone “*On the Fabric of the Human Body*” that “Of all the constituents of the human body bone is the hardest … intended to be like the foundation for the whole body … to perform the same function as do walls and beams in houses, poles in tents, and keels and ribs in boats.” In the 17th century, in his studies of motion Isaac Newton (1643–1727) wondered on the articulation of the thumb, and when knighted in 1705 elected for his coat of arms a pair of “shinbones” (tibia) crossed on a black background (Fig. 1) (Enlow, 1963; Grob, 2014). As late as the 19th century the German poet Johann Goethe (1749–1832) boasted of his superior knowledge of bones, “I know the bones of my fingers by heart” and ventured into osteology in his “*The Structure of Humans as Evidence of their Past*”.

Anecdotes and allegories notwithstanding, archeological and anthropological evidence indicates that whereas bone deformities were noted, and its fractures treated from time immemorial, their study remained essentially superficial and treated with benign neglect. Interestingly, it was corrective surgical attempts of bent bone childhood deformities that gave rise to the term “*orthopedics*” (from Greek for *ortho* = straight, *paedia* = children) first used in a 1741 French treatise on bone surgery by Nicolas Andry (1658–1742) titled “*L'Orthopédie*” (Orthopedics), one of the earliest disciplines to delve into osteology (Böni, 1999; Ijpma et al., 2012). It was about the same time that a scientific text on another ossified organ, teeth, was published in 1746 by another French surgeon Pierre Fauchard (1678–1761) as “*Le Chirurgien Dentiste*” (The Surgeon Dentist) hence also the origin of the terms “*dentiste*” and “*dentistrie*” (dentistry), derivatives from the French of the word “*dent*” for teeth (Enlow, 1963). Since then, there has been steady progress in the study of tissue calcification with remarkable advances achieved since 1950s (Fig. 1, Table 1). In the process of its conceptual evolution from a passive scaffold to a dynamically regulated organ with homeostatic functions the understanding of bone biology has undergone more revisions and paradigm shifts than that of any other organ.

**Table 1:** Number of PubMed entries for each listed subject in 1950 and 2020.

Subject	1950	2020
Bone	3003	64,489
Calcium	221	21,243
Phosphate	168	14,407
Parathyroid hormone	11	1570
Vitamin D3	4	1548
FGF23	0	403
Renal osteodystrophy	7	143
Osteoporosis	32	5544

This review explores the origins and conceptual evolution of how the once rather passive structural supporting role of bones of old unfolded into that of an increasingly complex organ with vital equipoise functions, particularly in its relationship with the kidney with which it is intimately linked in health and disease. To appreciate how it all began it is useful to reverse the path followed by a founder of nephrology Homer Smith (1895–1962), who after his 1951 magisterial “*The Kidney. Structure and Function in Health and Disease*” (Smith, 1951) went on to trace the evolutionary history of the kidney in his erudite 1953 book, “*From Fish to Philosopher*” (Smith, 1953). This recounting of kidney development foretells the story of bone development which also begun with that of fishes (Stubin, 2009; Donohue and Sansom, 2002).

## 2 Origins. A remembrance of things long past

It all goes back to countless million of years ago (Mya) when the changing climate and shifting tectorial plates of cooling grounds shaped the vicissitudes of evolving life on the planet earth. In what may well be over 3 billion years ago, as single cell organisms were evolving, calcium acquired an important role in cellular function as an intracellular signaling agent, which some 800 Mya was inscribed in chromosomes and in evolutionary happenstance transmitted in increasing complexity to their multicellular descendants (hydra, jellyfish) that emerged around 600–700 Mya (Knoll and Caroll, 1999). The critical period of interest for bone and by extension of calcium metabolism however was shaped well after multicellular life had emerged, specifically during the 60.3-million-year span of the so-called Devonian Period (419.2–358.9 Mya) of the Paleozoic Era (541–252 Mya), when fish moved back and forth from sea water to fresh water, to brackish streams and on to dry land, which is why this stretch of ancient times is also referred to as the Age of Fishes. This was a period of dramatic atmospheric shifts and mass extinctions necessitating considerable metabolic adaptive changes in the emerging species to transition from water to land (Gee, 2021). That is when tissue mineralization began to provide a protective exoskeleton and the preying teeth of emerging jawed predatory species (Knoll and Caroll, 1999; Smith and Hall, 1960; Stubin, 2009; Wagner and Aspenberg, 2011). Of relevance to the future discovery of the role of leptin in bone metabolism, this is also when ossification became coupled with the need to control appetite depending on availability of food in a changing violent external environment (Confavreux, 2011; Upadhyay et al., 2015). And, as fins began to change into the rudimentary limbs of arthropods migrating on land it marked the start of mineralization of internal collagen tissues as the elementary bones of the endoskeleton of the forthcoming future vertebrates. It is then that the mineral fraction of bones consisting mainly of calcium carbonate (calcite) used to build marine exoskeletons was replaced by the easier to precipitate and more insoluble hydroxyapatite crystals of calcium phosphate that bestowed bones with the greater stability and strength needed for mobility on land (Kawasaki, 2011; Monteiro et al., 2016). The hydroxyapatite content of bone (about 60–66 %) is higher in teeth (about 70–80 % in dentin) and even higher (about 89 %) in their outer enamel layer which is what makes teeth the hardest parts of the body and the ones best preserved in recovered archeological skeletons (Enlow, 1963; Grob, 2014). It is the stability of bones that has been the bread and butter of archeologists and preserved the record of evolutionary genetics that is currently helping decipher the family tree of human evolution (Pääbo, 1998). It was also in the Devonian Period that the regulatory gene network of bone homeostasis essential to vertebrate phylogeny developed, evolved, and increased in complexity in the evolving vertebrate species including the first identification of a role in mineral homeostasis by osteocytes emerged (Bianco, 2015; Bobewald, 2011; Haridy et al., 2021). Of note, it was the sediment of calcium carbonate of the microscopic shales of archaic planktonic organisms that some 100 Mya formed the White Cliffs of Dover (Monteiro et al., 2016).

With migration back and forth from water to land there emerged the vertebrates (400 Mya), the first mammals (200 Mya), the first hominids (6–7 Mya) and the first *Homo sapiens* (300 thousand years ago) necessitating metabolic adaptations during the journey from the calcium-rich environment of sea water (10–12 mM), to the less so of fresh water (0.5–1 mM) and none in the arid terrestrial environment (Doherty et al., 2015; Gee, 2021; Kawasaki et al., 2004; Williams, 2016). In the process, the metabolism of calcium changed from that of an abundant rejected waste product to one of a rare vital mineral absorbed in the intestines, preserved by the kidneys, and stored in bones. It is then that the genes of the regulatory network underlying tissue mineralization and calcium homeostasis were refined, with increasing complexity and precision in controlling the calcium balance of land-based species (Doherty et al., 2015; Peacock, 2010). As a result, the evolution of bone was paralleled by the development of a homeostatic biofeedback system that allowed for the precise control of bone mineral balance within which the parathyroid gland and vitamin D play a key role (Bickle, 2011; DeLuca, 2014). And so it was that the parathyroid encoding genes in the gills of fish evolved into the ectodermal pharyngeal pouch of mammalian embryonic life wherein the parathyroid glands are formed (Okabe and Graham, 2004). While the precursors of a pre-hormone ergosterol (misnamed a vitamin) originated in the cell membrane of the minute organisms of the plankton family, with the further evolution of its metabolism into ergocalciferol (vitamin D2) and cholecalciferol (vitamin D3) during the changes of life on earth that followed (Bickle, 2011; DeLuca, 2014).

It was also in the Devonian period that related fundamentals of life on land were established. Pertinent parallel evolutionary changes as amphibians evolved in the Devonian period were the rudiments of a respiratory system in the lungfish, known in Swahili as *Kamongo*, that Homer Smith sought, imported, studied, and narrated (Smith, 1932). It was also during this stretch of time when osmoregulation and excretion of nitrogenous products of migrating predatory fish led to the appearance of the beginnings of the vascular tufts of the glomerulus for the excretion of waste products by filtration, followed by increasing refinements in tubular regulatory function and the loop of Henle necessary for water, electrolyte, calcium, and phosphate regulation when life on land necessitated the preservation of water and scarce elements necessary to maintain homeostasis (Bochenbauer and Keita, 2021; Keogh et al., 2021; Koonan, 2012; Natochin, 1996; Schrier et al., 2004).

## 3 Landmark discoveries. Erecting the scaffold of osteology

As fundamental for survival as those evolutionary genetic transmutations of bone metabolism were, they went unknown until the past few decades. Until then, studies in bone metabolism were led by surgeons interested in repair of fractures. The study of bone began its transition from the observational descriptive discipline of old to that of an inquisitive scientific discipline that matured into the experimental investigation of bone in health and disease in the 17th century by anatomists, now armed with magnifiers, for its morphologic study and in the 18th century by experts in the emerging field of chemical analysis for its composition (Burr, 1989; Enlow, 1963; Grob, 2014), culminating in the 20th century in molecular studies that deciphered the written record of its genetic language summarized above (Fig. 1).

### 3.1 Structure

**Fig. 2:**
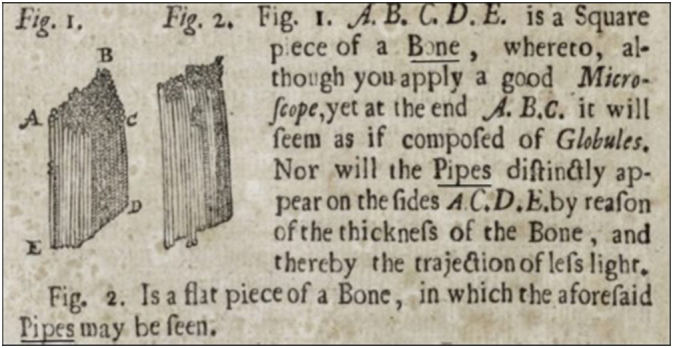
Etching depicting bone as rendered by Antonie van Leeuwenhoek (1632–1723) in 1678.

Reproduced from Leeuwenhoek (1678).

The structure of bone began to be explored by one of the very first microscopists, the self-taught Dutch scientist Antonie van Leeuwenhoek (1632–1723) who in 1674 began reporting his microscopic observations in letters to the Royal Society of London. In a 1678 letter, he described and illustrated cylindrical arrays of bone lamellae that he termed “*pipes*” as shown in Fig. 2 (Leeuwenhoek, 1678) which would unfold into the “*canals*” and “*canaliculi*” of the British physician Clopton Havers (1657–1702) in his 1691 book “*Osteologia Nova*” (Some New Observation on Bone) (Burr, 1989; Enlow, 1963; Grob, 2014); which were illustrated in 1857 by William Bowman (1816–1892) as the eponymous “*Haversian System*”, that would be determined to compose the basic functional unit of bone, the “osteon” in 1914 by the Jena physiologist Wilhelm Biedermann (1852–1929) (Burdon-Sanderson and Hulke, 1892; Burr, 1989).

**Fig. 3:**
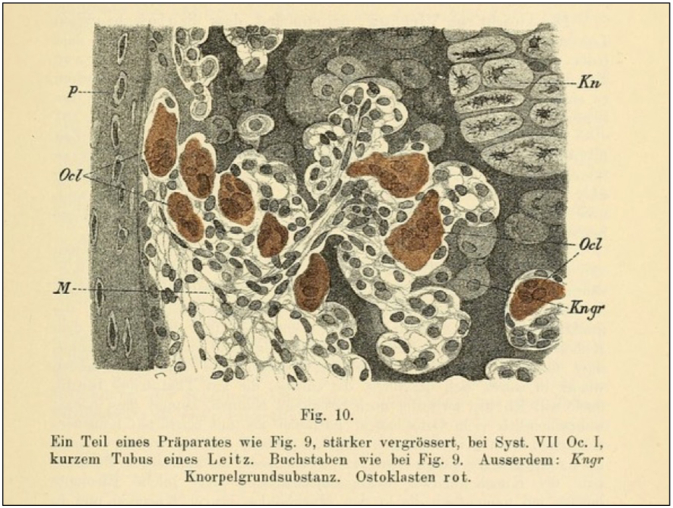
Cellular composition of bone as rendered by Albert von Kölliker (1817–1905) in 1873. Kn = cartilage of epiphyses; Kngr = cartilage matrix; M = medullary cavity; Ocl = osteoclasts; P = end of periosteal deposit of epiphysis.

Reproduced from Reference Kölliker (1899, page 319).

It was in the observation of nucleated cells (likely osteoblasts) in embryonic notochordal cartilage that the German physiologist Theodor Schwann (1810–1882) would begin to formulate his cell theory in 1838 (Fig. 1). That was the period when histology flourished, and it was in his studies of the cellular composition of various organs that the Swiss-German morphologist Albert Kölliker (1817–1905) described the cellular structure of bone in 1873, notably that of the large multinucleated cells he termed “*osteoklasten*” (osteoclasts) which theretofore were known by their French name of “*myéloplaxe*” (Fig. 3) (Kölliker, 1899). Of interest, Kölliker was a student of Jakob Henle (1809–1885) and a friend and admirer of William Bowman, which likely accounts for the chapter on bone in “*The Physiological Anatomy and Physiology of Man*” Bowman co-authored in 1857 with his mentor at King's College, Robert Todd (1809–1860) (Bianco, 2015; Bobewald, 2011; Burdon-Sanderson and Hulke, 1892; Burr, 1989; Kölliker, 1899).

It was also then that X-ray first became available for the study of bone, and Kölliker was one of the very first to have his hand publicly radiographed by his Würzburg colleague Wilhelm Röntgen (1845–1923) during his introductory lecture on radiology on January 23, 1896. And it was one of Kölliker's Würzburg students Karl Gegenbaur (1826–1903) who in 1864 identified the mononuclear bone cells he termed “*knochenbilden*” (bone-forming) cells or “*osteoblasten*” (osteoblast) that were gradually transitioned into the dendritic cells enclosed in the lacunae-canalicular cavities of the bone substance to become the “bone cells” that would be termed “osteocytes” (Fig. 1) (Kölliker, 1899; Robling and Bonewald, 2020). The extensive interconnected network of osteocytes was first described by Friedrich von Recklinghausen (1833–1910) who hypothesized that these cells may be capable of removing their surrounding bone minerals (Bobewald, 2011; Robling and Bonewald, 2020). It would be another half a century before technological advances in bone microscopy and recently of isolated bone cell cultures to understand their function. Specifically, the transition of early morphological observations to the dynamics of bone cells elucidating that osteoblasts deposit, osteoclasts destroy, and osteocytes coordinate bone modeling and remodeling in response to local cytokines, systemic hormones, and mechanical transduction to maintain the robust but delicately coordinated structure of bones (Bianco, 2015; Bobewald, 2011; Robling and Bonewald, 2020). And another several decades to document that while osteoblasts and osteocytes are derived from mesenchymal progenitors, osteoclasts are of hematopoietic origin (Bianco, 2015; Bobewald, 2011; Kawasaki et al., 2004).

### 3.2 Composition

The chemistry of bone mineralization was a relatively later development that began to be explored late in the 18th century (Sharma et al., 2020). In his classic 1733 “*Osteographia*”, the English surgeon William Cheselden (1688–1752) notes that “bones grow by degrees to harden” by the addition of “ossifying juices” or a “matter” that is “secreted into them” from blood. In his 1691 “*Osteologia Nova*” Havers had attributed bone accretion to the deposition of “terrestrial matter”. The French attorney turned chemist considered the founder of the Chemical Revolution, Antoine Lavoisier (1743–1794) reported in 1774 on the composition of bone ‘matter’ as that of ‘*chaux*’ from the Latin of ‘*calx*’ for lime (calcium salts). He went on to identify phosphorus as an element in 1777, after the Swedish chemists Johann Gottlieb Gahn (1745–1818) and Carl Wilhelm Scheele (1742–1786) had isolated elemental phosphorus from bone in 1771. Metallic calcium was obtained from bone in 1808 by Humphry Davy (1778–1829) confirming the prevailing notion of calcium phosphate crystals as the principal mineral constituent of the bone “matter” of old. In 1786, the deposited calcium orthophosphate crystals were termed “hydroxyapatite” (from Greek *apatáö* = I deceive) by the German geologist Abraham Werner (1749–1817) because they had been mistaken for so long as minerals other than calcium phosphate (Dorozhkin, 2013; Eliaz and Metoli, 2017; Enlow, 1963).

### 3.3 Disease

The first bone disease to be identified as a new illness was that of the crippling deformities of advanced rickets by the English physician Daniel Whistler (1619–1684) in his 1645 graduation thesis from Leyden University; and popularized shortly thereafter in a detailed 1650 report (*De Rachitide*) by Francis Glisson (1597–1677) (Elder and Bishop, 2014; Gibbs, 1994). It was originally reported as a disease of English children (*De morbo puerile Anglorum*) from Southwest England in the counties of Somerset and Dorset, which interestingly border on the county of Devon after which the Devonian Period discussed above was named in 1830s. Although rickets was noted as the most common cause of childhood deformities, and its occurrence in adults (*rachitis tarda*) was reported in 1723 as “*osteomalcia*” (from the Greek *osteo* = bone and *malakos* = soft) by the French surgeon Jean Louis Petit (1674–1750), it remained a disease of limited interest (Dorozhkin, 2013; Gibbs, 1994; Elder and Bishop, 2014; Enlow, 1963). It was as “*renal rickets*” that a link of bone to kidney disease was first reported in 1883 by a renal surgeon from Guy's Hospital, Richard Clement Lucas (1846–1915), 56 years after Richard Bright (1789–1858) had reported his eponymous disease from the same institution (Lucas, 1883). Lucas' prescient comment that “… late rickets and albuminuria are too frequently connected to be matters of chance …” would prove correct. The link, then variously reported as “renal dwarfism”, “renal infantilism” and “renal nanism” was expanded beyond that of rickets to include the lesions of “osteitis fibrosa cystica” (so-called von Recklinghausen's disease) termed “renal osteitis fibrosa cystica” in 1936 and “azotemic hyperparathyroidism” in 1937 by Fuller Albright (1900–1969) (Elder et al., Gibbs, 1994). With varied forms of bone lesions then being reported in chronic kidney disease (CKD) it was proposed in 1942 to term them collectively “*renal osteodystrophy*” by Shih-Hao Liu (1900–1974), a founder of Chinese endocrinology who had trained at the Rockefeller Institute with Donald van Slyke (1883–1971) (Fig. 1) (Liu and Chu, 1943). The term “*osteodystrophy*” (*osteo* = bone and *dystrophy* = degeneration) had been introduced in 1905 by the Polish surgeon Jan Mikulicz (1850–1905) to replace the term “*osteopathy*” that had been in use theretofore since the 1850s (Elder and Bishop, 2014; Gibbs, 1994).

Ultimately, it was the study of rickets that would begin to unravel the mysteries of bone metabolism in general and then of renal osteodystrophy that would reveal the intertwined complex relationship of bones and kidneys (Dent, 1970).

## 4 Rickets. The path to elucidating renal osteodystrophy

Endowed with its Latin medical name of ‘*rachitis*’ (a term from the Greek *rhakis* = spine, reflecting the clinical prominence of the curvature of the spine that also rhymes with rickets) bestowed by Francis Glisson, rickets became a formal disease deserving of investigation (Fig. 1). Over the following two centuries it became evident that contrary to its initial reports rickets was not just an English disease but one of global occurrence, and not a rare disease but one of increasing prevalence of epidemic proportions in developing industrial smoggy cities worldwide. Attributed from the outset to the damp cold climate of southwest England, fresh air was an early recommended treatment. Also, considered an illness of nutritional deficiency a host of supplements were suggested for its therapy eventually identifying cod liver oil as an effective remedy in the 1820s. As a result, by the second half of the 19th century prominent physicians, such as Armand Trousseau (1801–1869) in Paris, were promoting the unity of rickets and osteomalacia and recommending the use of cod fish oil and sunlight for their cure. Their effectiveness however continued to be debated given the heterogenous metabolic bone derangements which clinically simulate rickets but were not yet identified (Paget's disease, hyperparathyroidism, renal phosphaturia, osteogenesis imperfecta). As a disease of infants (ages 6 months to 3 years) the pathology of rickets was attributed to failure of lime salt (calcium phosphate) deposition in the osteoid tissue of growing bones (Dent, 1970; Elder and Bishop, 2014; Gibbs, 1994).

It was in the opening decades of the 20th century that experimental studies begun to decipher the pathophysiology of bone mineralization, which would result in two Nobel Prizes (Enlow, 1963; Grob, 2014). The first in Physiology and Medicine granted in 1903 to the Danish physician Niels Ryberg Finsen (1860–1904) “*in recognition of his contribution to the treatment of diseases, especially lupus vulgaris, with concentrated light radiation*”. The reason for the effectiveness of sunlight and phototherapy in rickets remained a mystery until nutritional studies of the period revealed that in addition to the essential dietary factors (protein, carbohydrate, fat, minerals) considered theretofore, there were also other necessary “accessory factors” or “vital amines” leading to the term “*vitamines*” that was formally abbreviated to “vitamins” in 1920. That paved the way to identifying the mysterious fat soluble “anti-rachitic factor” of cod fish oil as the fourth vitamin (D) by the American biochemist Elmer McCollum (1879–1967) and his associates in 1922 (DeLuca, 2014; Dent, 1970; McCollum et al., 1922). Simultaneously, it was shown that exposure to ultraviolet light bestowed anti-rachitic properties to certain foods (oils, milk, cereals) that was inherent to their sterol content. This in turn led to the second Nobel Prize now in Chemistry to the German chemist Adolf Windaus (1876–1959) in 1928 “*for his research into the sterols and their connection to the vitamins*”. The mechanism was resolved by the 1940s when it was shown that it was ultraviolet sunlight exposure of the naturally occurring pre-hormone (7-dehydrocholesterol) in vertebrate skin that results in the photosynthesis of vitamin D_3_ (cholecalciferol) (Bickle, 2011; DeLuca, 2014). Extensive experimental and clinical studies that ensued then revealed that coincident with the improvement of rickets that followed replacement with vitamin D_3_ analogs there was increased intestinal calcium absorption, a rise in the levels of serum calcium and phosphorus, an initial drop followed by a rise of calcium excretion in the urine, and an increase in bone mineralization. As vitamin D_3_ metabolites began to be identified it became evident that their effectiveness in increasing calcium absorption and bone mineralization was enhanced by their increased polarity following hydroxylation first to 25(OH)D_3_ (calcidol) in the liver and then to its biologically active form 1,25(OH)_2_D_3_ (calcitriol) in the kidney (DeLuca, 2014; McCollum et al., 1922). Radioactive fluorescent studies led to the identification of receptors for the active form of vitamin D_3_ (VDR) in most tissues of the body, specifically in the intestine, kidney, and parathyroid glands.

The parathyroid glands in humans were discovered in 1880 by the Swedish anatomist Ivar Sandström (1852–1889), then a medical student at Uppsala (Eknoyan, 1995; Johansson, 2015). His report was rejected for publication by Rudolph Virchow in his *Archiv*, and the importance of the glands went barely noticed until 1908 when the Canadian pathologist William MacCallum (1874–1944) and his pharmacology associate Carl Voeglin (1879–1960) demonstrated that parathyroidectomy resulted in hypocalcemic tetany. Parathyroid hormone (parathyrin) was extracted from the glands by James Collip (1892–1965) in 1925 and subsequently isolated and purified by Howard Rasmussen (1925–2005) and his associates which then allowed for the experimental study of the effect of parathormone on bone, kidney, and intestines (Eknoyan, 1995; Rasmussen, 1961; Sharma et al., 2020). Coupled with the landmark studies of Fuller Albright in the 1930–1940s the vital role of the parathyroid glands in bone disease and calcium homeostasis were then established. As a result, when the functions of Vitamin D were being explored it became evident that parathyroid hormone (PTH) production and secretion were directly related to the level of circulating calcitriol (1,25(OH)_2_D_3_), wherein increased levels of PTH due to low calcium in the blood stimulated the renal production of calcitriol, and that the principal role of calcitriol was that of maintaining plasma calcium levels even at the expense of mobilizing bone calcium necessary to maintain the availability of this now ubiquitous signaling molecule by the increased levels of circulating PTH (Kleeman et al., 1969). It was these metabolic breakthroughs that would begin to unravel the pathophysiology of renal osteodystrophy.

## 5 Renal osteodystrophy. The path to elucidating bone metabolism

Much as identifying rickets had provided a rallying point for the study of bone metabolism in the opening decades of the 20th century, that of studying renal osteodystrophy now provided for its expansion into the broader biology of bone in health and diseases in the second half of the 20th century. This was the period when studies of renal physiology were flourishing and being applied to clinical medicine in that formative period of the discipline that would become nephrology in the 1960s. The catalytic factor that accelerated those studies and expanded their clinical application was the advent of maintenance dialysis in the early 1960s (Fig. 1).

Without detracting from the important contributions of several pioneer investigators of the bone lesions of renal osteodystrophy after 1940, the contributions of Sydney William Stanbury (1919–1996) of the Manchester Infirmary deserves recognition (Fig. 1). Prompted by his initial studies of renal tubular disorders, his 1950s metabolic studies of “azotaemic renal osteodystrophy” that followed were central to establishing the varied skeletal lesions of different pathogenesis which occur in patients with renal failure that presented with the common clinical features of metabolic acidosis, usually elevated serum phosphorus levels, variable serum calcium levels that were often low but occasionally elevated, and in advanced cases of bone disease elevated alkaline phosphatase levels (Stanbury, 1957). These were shown to be coupled with reduced levels of vitamin D_3_ and elevated levels of PTH that were variably responsive to phosphate restriction, calcitriol administration and partial parathyroidectomy (Kleeman et al., 1969; Scriver, 1979; Sherrard et al., 1993; Stanbury, 1957). The 1960 “intact nephron hypothesis” of Neal Bricker (1927–2015) and his associates provided key evidence for the crucial role of hyperphosphatemia in the genesis of the bone lesions that led to the dominance of controlling phosphate levels in CKD (Slatopolsky, 2011).

With life-saving maintenance dialysis in the 1960s came new bone lesions that were not previously recognized or encountered such as “aluminum bone disease”, “osteosclerosis”, vascular calcification, “tertiary hyperparathyroidism” and “adynamic bone disease”; while kidney transplantation and its therapy added yet another set of bone lesions specially that of increased fracture risk to levels higher than those of dialyzed patients due to corticosteroids (Drüeke and Evenepoel, 2019; Jørgensen et al., 2022; Malluche et al., 2011; Miller, 2014; Parfitt, 1998).

Technological advances in the histology of hard tissues, imaging, and chemical analysis allowed for the study of these new abnormalities in greater detail and precision revealing marked variability and overlap of the bone lesions encountered in individual patients with kidney failure. Increasing interest in renal osteodystrophy was paralleled by a broader interest in osteology prompted by the increasing prevalence of osteoporotic bone fractures in the growing elderly population worldwide (Moe, 2017; Ott, 2017; Patlak and Teitelbaum, 2001). As a result, studies in bone biology that had begun by bone surgeons early in the 17th century, then advanced by bone morphologists in the 17th and 18th centuries, and enriched by those of chemists in the 19th through the 20th century, now entered the domain of endocrinologist, renal physiologists, radiobiologists, and physicists that brought the understanding of bone physiology and metabolism in the 21st century to an unexpected prominence, particularly that of its regulatory role in homeostasis in general (Guntur and Rosen, 2012; McNermy and Nickolas, 2017; Karsenty and Ferron, 2012).

### 5.1 Bone Metabolism. From languid structural support to a dynamic participant in homeostasis

Apart from the noble quest for scientific knowledge that drove investigation, as was the case with the study of rickets and renal osteodystrophy that had driven inquiry into bone metabolism through the mid-20th century, it was the study of osteoporosis that provided the rallying point and stimulus of bone research in the closing decades of the 20th century that has continued to expand with increasing momentum into the 21st century (Grob, 2014; Patlak and Teitelbaum, 2001; Stride et al., 2013).

The knowledge that bones are not inert structures but change over time had long been a medical tenet that was clearly stated in 1790 by the Scottish surgeon John Hunter (1728–1793) as, “bone is constantly changing in its matter” and grows by “outer deposition and inner resorption”. The clinical features of altered bone metabolism with aging especially in women were known by the 19th century. In the chapter on femoral head fractures of his 1822 book “*A Treatise on Dislocations and Fractures of Joints*” Astley Cooper (1768–1841), an eminent English surgeon who was a tutor and friend of Richard Bright at Guy's Hospital, reported that “in advanced periods of life … rarely under fifty years of age … bones become thin in their shell and spongy in texture … soft from the absorption of their phosphate of lime … fragile … women are much more likely to this species of fractures than men.” This clinical description of a bone disease was established as a clinical diagnosis shortly afterwards in 1833 when it was termed “*ostéporose*” (from the Greek osteo = bone, and poros = passage or pore) by the French pathologist Jean Lobstein (1777–1835) (Fig. 1) (Grob, 2014; Patlak and Teitelbaum, 2001; Stride et al., 2013). But like other bone diseases of the period its clinical study went unexplored, while studies of bone modeling and their mechanical control continued by surgeons interested in fractures; notably those of the German surgeon Julius Wolff (1836–1902), the first professor of Orthopedic at the Charité Hospital in Berlin, who set the laws governing bone growth and remodeling in his 1892 magnum opus “*The Law of Transformation of the Bone*” (Wolff, 1892).

**Fig. 4:**
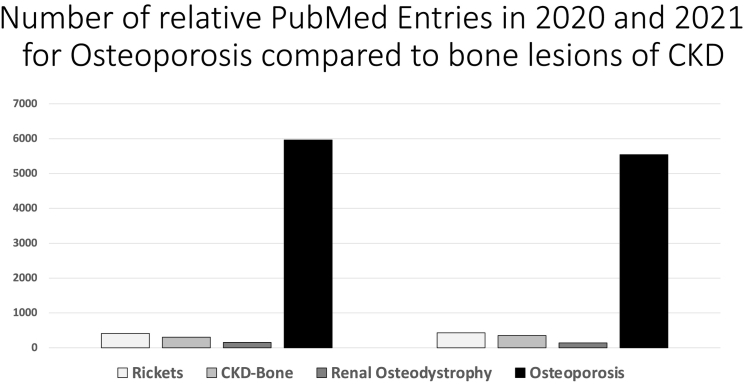
Number of PubMed entries for osteoporosis compared to those of other bone diseases in 2020 and 2021.

Osteoporosis was popularized decades after its identification as “post-menopausal osteoporosis” by Fuller Albright in the early 1940s (Fig. 1) (Grob, 2014). The systematic metabolic studies of bone diseases initiated by Albright then provided the framework for the integration of basic and clinical studies that would elucidate the understanding of bone function in health and disease that followed (Axelrod, 1970). Still, no one realized the epidemic that osteoporosis would become by the end of the century and is now considered the most prevalent skeletal disorder with a lifetime risk like that of coronary heart disease (Grob, 2014). As a result, studies in osteoporosis have grown exponentially since the 1980s, promoted by socio-political concerns of the rising levels of osteoporotic bone fractures in a growing segment of the elderly population and driven by clinical trials supported to a great extent by the pharmaceutical industry (Fig. 4, Table 1). A principal beneficiary of this has been the recent transformative changes in the understanding of bone cell biology, the cellular organization of bone development, the complexities of bone modeling and remodeling cycle, and the potential of their quantification by on-going technological advances (Robling and Bonewald, 2020; Confavreux, 2011).

The technological advances that have particularly shaped the understanding and evaluation of the architectural evolution of bone biology were histomorphometry and dual-energy x-ray absorptiometry (DEXA). Bone histomorphometry was prompted in the late 1950s when it was shown that tetracycline, a natural antibiotic produced by bacteria discovered in 1948, deposits in vivo at sites of bone formation that can be studied by fluorescent microscopy (Frost, 1969). By the 1980s research in its use allowed for the finer evaluation of bone from both a static and dynamic perspective (Sherrard et al., 1974). Recently, bone volume was added as an important measure in renal osteodystrophy resulting in the recommendation to quantify bone turnover, mineralization, and volume, the so-called TMV system (Moe, 2017). Parallel advances in radiobiological imaging techniques such as the development of DEXA and of high-resolution peripheral quantitative computed tomography which by their non-invasive nature have come to replace histomorphometry in most clinical studies (Dempster et al., 2013; Jee, 2005; Nicholas et al., 2010). The novelty of all those fields and their variable results necessitated a series of consensus conferences to establish the definition, terminology, measurement units, and their appropriate therapeutic targets all of which continue to be refined in consensus statements and guidelines (Dempster et al., 2013; Jee, 2005; Moe and Drüeke, 2006; Moe, 2017; Ott, 2017).

Much as structural studies elucidated the strength of bones in their fundamental function, that of its indispensable role in calcium and phosphate homeostasis has further improved the appreciation of bone as a dynamic organ. Extracellular and intracellular calcium is tightly regulated due to the importance of calcium in membrane potential and intracellular signaling. Calcium fluxes in and out of bone (which contains over 99 % of total body calcium) are necessary to accommodate this tight regulation. Bone calcium balance describes the net gain, loss, or equilibrium of calcium moving to and from bone. Dual calcium isotope methodologies have been utilized to quantify these fluxes and bone balance in response to therapeutic interventions but are expensive and involve clinical research center testing (Hill et al., 2013; Hill Gallant et al., n.d.). More recently advances in mass spectrometry have allowed the assessment of naturally occurring (in food and bone) stable calcium isotopes, allowing the development of techniques to assess bone balance from blood or urine samples (Shroff et al., 2021; Shroff et al., 2022). The possibility of measuring real time changes will almost certainly advance knowledge of the role of bone in normal mineral homeostasis.

The acceleration of molecular and genetic studies has identified a host of serendipitous messengers relevant to renal osteodystrophy, anemia, heart disease, metabolism and more. Notably that of the fibroblast growth factor-23 (FGF23), that suppresses renal phosphate reabsorption and calcitriol synthesis, and of its receptor α-Klotho of the family of enzymes involved in aging termed Klotho, after the Greek mythological figure of Fate who controlled the thread of life (Ketteler et al., 2018; Ott, 2017; Patlak and Teitelbaum, 2001). Additional hormones secreted from bone include uncarboxylated osteocalcin, that acts on the pancreas to increase insulin production, on fat to regulate metabolism, and on the testis to regulate male fertility; sclerostin that regulates insulin and adipose tissue; and lipocalin 2 that suppresses appetite (Lee, 2008; Takashi and Kawanami, 2022).

In summary, over the past 400 years it took to decipher the vital functions of this dynamic and critically essential organ, bone has gone from an underrated inert foundational structure performing “the same function as do walls and beams in houses…” to an intricate endocrine organ with a fundamental role in metabolism, kidney disease, vascular calcification, anemia, fertility, and obesity.

## CRediT authorship contribution statement

**Garabed Eknoyan** conceived, researched, and wrote a first draft of the article. Selected and prepared the figures and tables. **Sharon M. Moe** reviewed, edited, and finalized the article.

## Declaration of competing interest

The authors declare that they have no known competing financial interests or personal relationships that could have appeared to influence the work reported in this paper.

## Data Availability

No data was used for the research described in the article.
